# Application of Long-Chained Auxin Conjugates Influenced Auxin Metabolism and Transcriptome Response in *Brassica rapa* L. ssp. *pekinensis*

**DOI:** 10.3390/ijms25010447

**Published:** 2023-12-28

**Authors:** Ana Smolko, Jelena Repar, Marija Matković, Iva Pavlović, Aleš Pěnčík, Ondřej Novák, Jutta Ludwig-Müller, Branka Salopek-Sondi

**Affiliations:** 1 Department for Molecular Biology, Ruđer Bošković Institute, Bijenička Cesta 54, 10000 Zagreb, Croatia; ana.smolko@chem.pmf.hr (A.S.); jelena.repar@irb.hr (J.R.); 2 Department for Organic Chemistry and Biochemistry, Ruđer Bošković Institute, Bijenička Cesta 54, 10000 Zagreb, Croatia; marija.matkovic@irb.hr; 3 Laboratory of Growth Regulators, Faculty of Science of Palacký University & Institute of Experimental Botany of the Czech Academy of Sciences, Šlechtitelů 27, CZ-783 71 Olomouc, Czech Republic; nizic.iva@gmail.com (I.P.); ales.pencik@upol.cz (A.P.); novako@ueb.cas.cz (O.N.); 4 Institute of Botany, Technische Universität Dresden, Zellescher Weg 20b, 01062 Dresden, Germany; jutta.ludwig-mueller@tu-dresden.de

**Keywords:** amino acid auxin conjugates, *Brassica rapa*, indole-3-acetic acid, indole-3-butyric acid, indole-3-propionic acid, auxin metabolome, root growth inhibition, transcriptome

## Abstract

Auxin amino acid conjugates are considered to be storage forms of auxins. Previous research has shown that indole-3-acetyl-L-alanine (IAA-Ala), indole-3-propionyl-L-alanine (IPA-Ala) and indole-3-butyryl-L-alanine (IBA-Ala) affect the root growth of *Brassica rapa* seedlings. To elucidate the potential mechanism of action of the conjugates, we treated *B. rapa* seedlings with 0.01 mM IAA-, IPA- and IBA-Ala and investigated their effects on the auxin metabolome and transcriptome. IBA-Ala and IPA-Ala caused a significant inhibition of root growth and a decrease in free IAA compared to the control and IAA-Ala treatments. The identification of free auxins IBA and IPA after feeding experiments with IBA-Ala and IPA-Ala, respectively, confirms their hydrolysis in vivo and indicates active auxins responsible for a stronger inhibition of root growth. IBA-Ala caused the induction of most DEGs (807) compared to IPA-Ala (417) and IAA-Ala (371). All treatments caused similar trends in transcription profile changes when compared to control treatments. The majority of auxin-related DEGs were found after IBA-Ala treatment, followed by IPA-Ala and IAA-Ala, which is consistent with the apparent root morphology. In addition to most *YUC* genes, which showed a tendency to be downregulated, transcripts of auxin-related DEGs that were identified (*UGT74E2*, *GH3.2*, *SAUR*, *IAA2*, etc.) were more highly expressed after all treatments. Our results are consistent with the hypothesis that the hydrolysis of conjugates and the release of free auxins are responsible for the effects of conjugate treatments. In conclusion, free auxins released by the hydrolysis of all auxin conjugates applied affect gene regulation, auxin homeostasis and ultimately root growth inhibition.

## 1. Introduction

Auxins are one of the essential plant hormones important for plant growth and development. They regulate gene expression, cell division, proliferation and plant tissue differentiation. They are signaling molecules that control the expression of genes through interaction with the ubiquitin complex (regulatory particles) and with transcription factors, including auxin response factors (ARF) and AUX/IAA repressors. Members of the F-box TIR1/AFB family of proteins act as auxin receptors in a signaling cascade, which is a complex process and details may be found in recent reviews [1,2]. An optimal auxin concentration is necessary to regulate this network of processes, while high concentrations have an inhibitory and toxic effect [3,4,5]. Thus, the control of auxin levels, so-called auxin homeostasis, is crucial for proper plant development and is achieved through the processes of biosynthesis, transport, degradation and reversible and irreversible auxin conjugation [3,4,5]. In addition to indole-3-acetic acid (IAA), which was the first discovered and is the most abundant natural auxin in the plant kingdom, 4-chloroindole-3-acetic acid (4-Cl-IAA) and phenylacetic acid (PAA) are well-known natural auxins but may not occur in all plant species [1]. The long-chained auxins indole-3-butyric acid (IBA) and indole-3-propionic acid (IPA) are also considered to be natural auxins.

IBA was originally discovered as a synthetic compound that induced root initiation in a variety of plants [6]. IBA–amino acid conjugates were reported as rooting agents in blueberries [7]. IBA and IPA as well as their amino acid conjugates showed strong root growth inhibition in a root growth assay [8], confirming their auxin activity. IBA has been identified as an endogenous compound by gas chromatography–mass spectrometry in a variety of plants, including pea, cypress, maize, carrot, tobacco and Arabidopsis [9]. However, Novák et al. [10] found no evidence of IBA in several plants, but this could be a consequence of extraction and/or cultivation methods. The auxin activity of IBA in several plants was suggested to be the result of its conversion to IAA through a multi-step process similar to fatty acid β-oxidation [11,12]. The literature suggests that plants use distinct carriers for IBA and IAA transport. The carrier AUX1 acts as an influx carrier for IAA, but not IBA. PINs and ABCBs act as efflux carriers for IAA, but not for IBA. Conversely, the PDR family proteins ABCG36 and ABCG37 appear to efflux IBA, but not IAA [13]. These independent transport systems may provide a mechanism to specifically move an inactive precursor, thus avoiding auxin responses during transport [6].

The physiological role of IPA in free and conjugate forms in plants is still an enigma. IPA was initially discovered by GC-MS in the hypocotyls of *Cucurbita pepo* L. [14], and subsequently in the roots of *Pisum sativum* L. seedlings [15]. IPA is one of the secondary metabolites identified by Walker et al. [16] in the root exudate of Arabidopsis upon treatment with salicylic acid. The latter is often produced by plants in reaction to microbial attack. It is well known that microorganisms such as *Clostridium* species [17] and ruminal bacteria [18] are able to produce IPA from tryptophan. Barkawi et al. [19] could not detect IPA in tomato and Arabidopsis, although they developed a reliable, sensitive and robust method for the purification and quantification of IAA and other auxins from plant tissue. Thus, the existence of IPA as a potential endogenous auxin and its physiological role remain questionable.

It is well known that auxins are active in their free forms and can be stored or deactivated as auxin amide- and ester-conjugates [4]. The physiological activity of auxin amide-conjugates has been examined by numerous bioassays [3,8]. It is suggested that the auxin activity of conjugates is indirect and results from endogenous hydrolysis and the release of free auxin forms. However, some recent investigations suggest a direct signaling function of conjugates in various processes. For example, IAA-Asp showed mitigating effects against salinity and metal stress in peas [20], and IAA-Ile had a protective role in the abscission zone of tomato flowers [21].

The main enzymes involved in the hydrolysis of amide-conjugates are auxin amidohydrolases (ILR, ILLs, IAR), previously characterized in *Arabidopsis thaliana*, *Medicago truncatula*, *Triticum aestivum* and *Brassica rapa* [8,22,23,24,25,26]. The hydrolyzing activity of auxin amidohydrolases acting on long-chain auxin–amino acid conjugates IPA-Ala and IBA-Ala was first reported for a *T. aestivum* auxin amidohydrolase (TaIAR3) [23], then for *M. truncatula* [24] and for *B. rapa* [8]. In the latter work, it was shown that the velocity of the hydrolyzing reaction of BrILL2 decreased in the order IPA-Ala > IBA-Ala > IAA-Ala, which agrees with a root growth bioassay showing higher growth inhibition caused by IPA-Ala and IBA-Ala compared to IAA-Ala.

Our hypothesis is that IAA-Ala, IPA-Ala and IBA-Ala treatments affect gene expression, in particular the genes related to auxin metabolism as well as auxin metabolome, and consequently lead to root growth inhibition. Thus, we performed feeding experiments with IAA-Ala, IPA-Ala and IBA-Ala using *B. rapa* seedlings and examined the auxin metabolome and transcriptome in the treated seedlings. The specific tasks were: (1) to examine whether endogenous auxin-amidohydrolases were able to hydrolyze long-chained auxin conjugates, as was shown previously by in vitro assays [8,23,24]; (2) to identify genes affected by treatments and correlate them, if possible with metabolites and root growth inhibition, and (3) to obtain information about the mode of action of auxin conjugates (whether they act directly or indirectly by free auxins released through the hydrolyzes process of conjugates).

## 2. Results

### 2.1. Root Growth Bioassay

The amino acid conjugates IAA-Ala, IBA-Ala, IPA-Ala at a concentration of 10 µM in one percent agar plates were tested for their inhibitory effect on the primary root growth of Chinese cabbage (*B. rapa*) seedlings. Based on our previous results of treatments performed at concentrations 0.0001–0.1 mM, we decided to use herein 0.01 mM concentration, which showed moderate inhibition of root growth. Auxin-free agar plates were used as a control. The resulting growth of the primary root (PR) is shown in Figure S1. Of all auxin conjugates, IBA-Ala caused the highest inhibition of root growth under the experimental conditions used (61.62% PR growth compared to the control), followed by IPA-Ala (74.26% PR growth compared to the control), while IAA-Ala did not cause any significant PR growth inhibition at the concentration used (98.20% PR growth compared to the control).

### 2.2. Analysis of Auxin, Auxin Precursors and Metabolites

**Figure 1:**
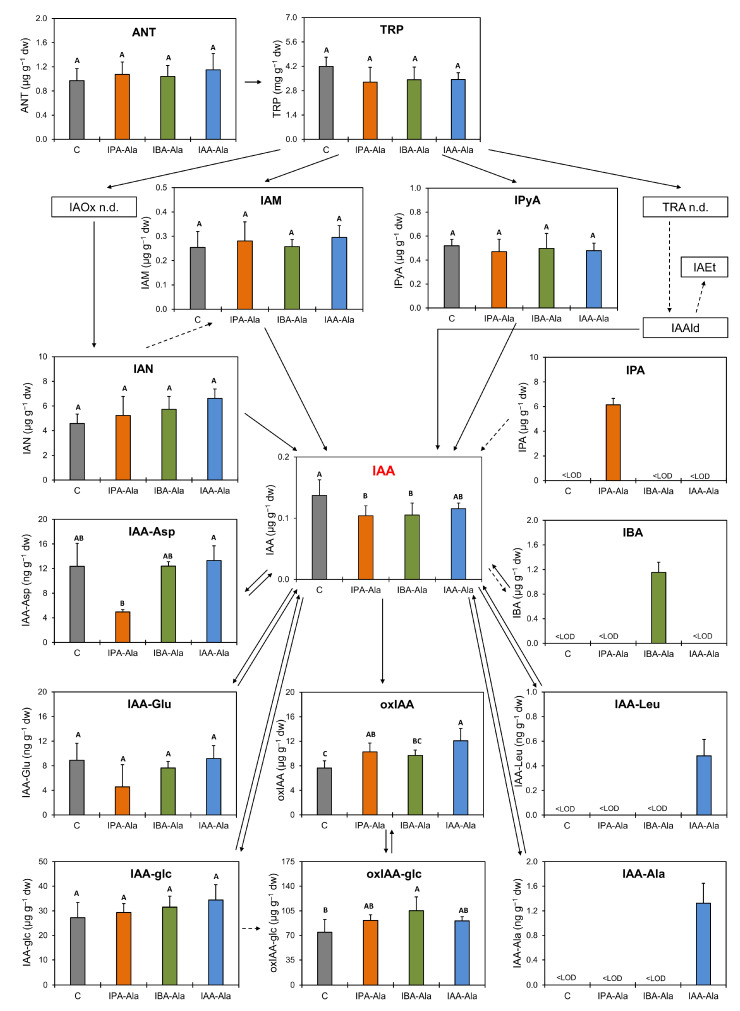
Changes in auxin metabolism in *B. rapa* seedlings after 24 h treatment with auxin conjugates (IBA-Ala, IPA-Ala and IAA-Ala c = 10 µmol µmol L^−1^) and untreated control (C): indole-3-acetic acid (IAA), indole-3-propionic acid (IPA), indole-3-butyric acid (IBA); biosynthesis precursors: anthranilate (ANT), tryptophan (TRP), indole-3-acetamide (IAM), tryptamine (TRA), indole-3-acetaldoxime (IAOx), indole-3-acetaldehyde (IAAld), indole-3-acetethanol (IAEt), indole-3-pyruvic acid (IPyA), indole-3-acetonitrile (IAN) and metabolites: 2-oxoindole-3-acetic acid (oxIAA), indole-3-acetyl-L-aspartate (IAA-Asp), indole-3-acetyl-L-glutamate (IAA-Glu), indole-3-acetyl-L-leucine (IAA-Leu), indole-3-acetyl-L-alanine (IAA-Ala), indole-3-acetyl-1-O-ß-d-glucose (IAA-glc) and 2-oxoindole-3- acetyl-1-O-ß-d-glucose (oxIAA-glc). Results are expressed in µg g^−1^ dry matter (dw) or in ng g^−1^ dry matter for each treatment (n = 4). Statistically significant differences in treatments (IBA-Ala or IPA-Ala or IAA-Ala; c = 10 µmol L^−1^) compared to untreated control (C) (=are shown in different letters with a significance level of 0.05 (*p* <0.05) in the ANOVA test with Tukey HSD (honest significant difference) post hoc statistical significance test). <LOD—below the limit of detection, n.d.—not determined. Solid arrows indicate steps catalyzed by known enzymes [10,27,28,29,30,31]. Dashed arrows indicate steps that may involve multiple enzymes and have not been well defined.

Auxin precursors and metabolites were identified and quantified by high performance liquid chromatography coupled with mass spectrometry (UHPLC-MS/MS). In *B. rapa* seedlings, free IAA, auxin precursors and metabolites, as well as auxin conjugates, for which internal standards existed at the time of the study, were quantified (Figure 1). After treating *B. rapa* seedlings for 24 h with any of the three conjugates (10 µM IAA-Ala, 10 µM IPA-Ala or 10 µM IBA-Ala, respectively), the content of the auxin biosynthetic precursors anthranilate (ANT), tryptophan (TRP), indole-3-acetamide (IAM), indole-3-pyruvic acid (IPyA) and indole-3-acetonitrile (IAN) had not changed significantly. The amount of Trp appeared to be present in milligram quantities in seedlings after all treatments, while all other precursors tested were measured in the microgram range. The levels of the detected free forms of the auxins IPA, IBA and IAA were approximately 6 µg g^−1^ dry dw, 1.2 µg g^−1^ dw and 0.12 µg g^−1^ dw, respectively, in the IPA-Ala, IBA-Ala and IAA-Ala treatments. The auxins IPA and IBA were exclusively detected in feeding experiments with IPA-Ala and IBA-Ala, respectively, and therefore are direct evidence for the hydrolyzes of the corresponding conjugates IPA-Ala and IBA-Ala in vivo. The content of free IAA was significantly reduced in the treatment with IPA-Ala and IBA-Ala, but not in the treatment with IAA-Ala (Figure 1). The content of the catabolic pathway precursors IAA-Asp and IAA-Glu did not change significantly in the treated seedlings compared to the control (Figure 1), while the oxIAA content increased significantly in the treatment with IPA-Ala and IAA-Ala, whereas the oxIAA-glc content increased significantly only in the treatment with IBA-Ala. The amide-conjugate IAA-Leu was only identified in the treatments with IAA-Ala (1.32 × 10^−3^ ± 0.33 × 10^−3^ ng g^−1^ dw). Unhydrolyzed IAA-Ala was detected in IAA-Ala-treated seedlings. However, potentially unhydrolyzed IBA-Ala and IPA-Ala were not identified, probably due to methodological limitations.

### 2.3. RNA-Seq Analysis

#### 2.3.1. Overall Changes in Gene Expression upon Treatments

**Figure 2:**
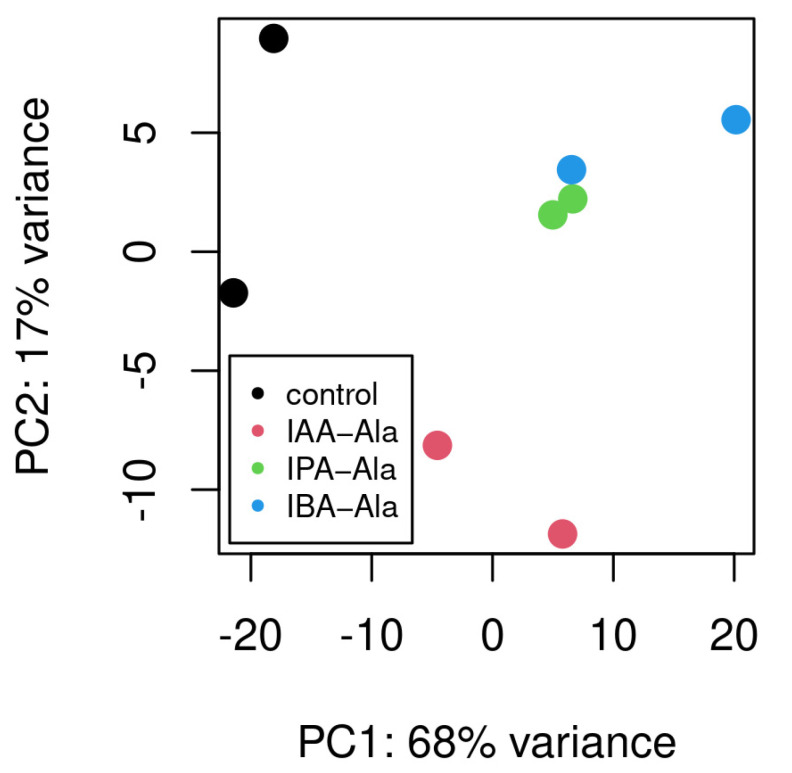
PCA plot on rlog-transformed sample gene counts. Rlog function from Deseq2 package transforms the count data to the log2 scale through a method which minimizes differences between samples for rows with small counts and which normalizes with respect to library size. Different treatments are expected to map to different parts of a PCA plot. PC1—first principal component, PC2—second principal component.

After treatment of *B. rapa* seedlings with IAA-Ala, IPA-Ala and IBA-Ala, total RNA from two biological replicates was sequenced and analyzed. The read counts per gene were estimated for each replicate and compared to the untreated control. The PCA analysis of read counts per gene and per treatment shows specific groupings for the control and IAA-Ala replicates. When comparing the IPA-Ala and IBA-Ala replicates to the controls and IAA-Ala treatments, the former group closely together, suggesting that there is a general similarity in gene expression following these treatments (Figure 2). After treating *B. rapa* seedlings with IAA-Ala, IPA-Ala and IBA-Ala, we identified 371, 417 and 807 differentially expressed genes (DEGs), respectively (Tables S1–S3). The number of DEGs did not correlate with the total number of uniquely mapped reads (on average 2.31, 2.09 and 2.27 × 10^7^, respectively). Therefore, IBA-Ala treatment altered the gene expression of almost twice as many genes as IAA-Ala and IPA-Ala treatments, suggesting that it is the strongest effector among the conjugates used.

#### 2.3.2. Differentially Expressed Genes (DEGs) across Gene Ontology (GO) Categories Linked to Treatments

**Figure 3:**
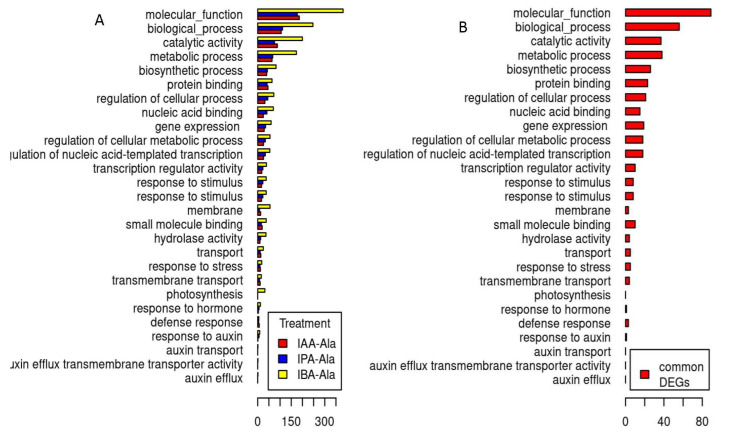
DEG counts per treatment per selected GO categories (**A**) and counts of DEGs common to all three treatments per selected GO categories (**B**). One gene may belong to several GO categories. Only GO categories with 30 or more genes are included in the figure. The distribution of DEGs across all the annotated GO categories and their ancestor nodes are available in Figure S1.

**Figure 4:**
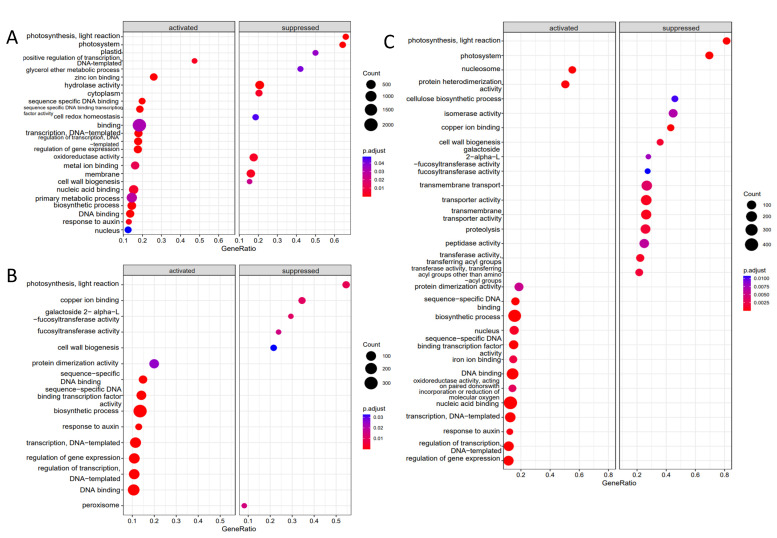
Dotplot showing GO categories that are significantly enriched (GeneRatio > 0.5) or depleted (GeneRatio < 0.5) of DEGs after the IAA-Ala (**A**), IPA-Ala (**B**) and IBA-Ala treatment (**C**) according to the GSEA analysis.

The distribution of DEGs across selected GO categories is shown in Figure 3A (the distribution of DEGs across all the annotated GO categories is available in Figure S2). The distribution of DEGs across GO categories is similar after all three treatments, indicating a similar qualitative effect. The quantitative differences, i.e., the higher number of affected genes after the IBA-Ala visible in Figure 3A, is in line with the higher number of DEGs detected after this treatment. The number of DEGs per category reflects in part the total number of genes that can potentially belong to the category, so categories with a large numbers of genes are expected to contain proportionally more DEGs. To compare whether some GO categories are disproportionately affected by the treatments, we performed a GSEA analysis (Figure 4).

The GO category of photosynthesis may represent an exception to the general patterns in the distribution of DEGs across GO categories following the three treatments, since no DEGs were detected after the application of IAA-Ala and IPA-Ala, as opposed to the IBA-Ala treatment (Figure 3A). In contrast to this observation, the effect on photosynthesis is detectable in the DEG-calls-independent GSEA analysis of all three data sets (Figure 4). Here, a disproportionately large number of photosynthetic genes were found at the lower level of the logFC ranking of genes, demonstrating a negative effect of IAA-Ala, IPA-Ala and IBA-Ala on the expression of photosynthetic genes. In addition, IBA-Ala affected more GO categories (activated and repressed) than IAA-Ala and IPA-Ala (Figure 4). All treatments activated the genes in the GO category “response to auxin”.

The three treatments differed only partially in the genes they affected. In total, 193 genes were common DEGs, i.e., genes that changed their expression after all three treatments (Figure S3). The changes in gene expression generally had the same direction (activation or repression) after different treatments compared to the control. The distribution of common DEGs across GO categories is similar to the distribution after the three treatments (Figure 3A,B). Overall, there seems to be a remarkable similarity between the effects of IAA-Ala, IPA-Ala and IBA-Ala on gene expression, as well as on the phenotype of *B. rapa* seedlings. A remarkable number of common DEGs are heat shock and other stress response proteins, a result consistent with the detrimental effects of the treatments on phenotype.

The 100 DEGs with the strongest changes in gene expression following treatments with IAA-Ala, IPA-Ala and IBA-Ala are shown in Figures S4–S6, respectively. Again, a similar direction (activation or repression) of the three treatments compared to the control can be seen, with different intensities of over- and under-expression, depending on the treatment.

#### 2.3.3. DEGs Linked to Auxin Metabolism

**Figure 5:**
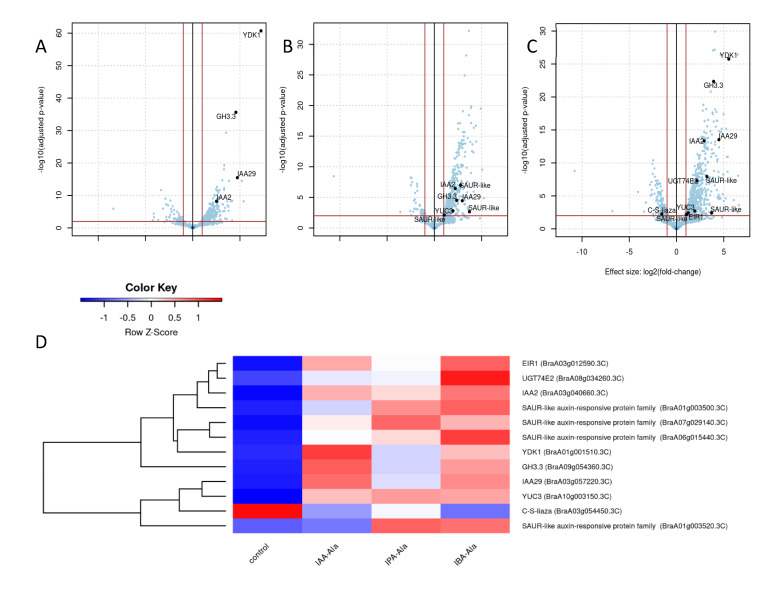
DEGs involved in auxin metabolism. Volcano plot of genes after IAA-Ala treatment (**A**), IPA-Ala treatment (**B**) and IBA-Ala treatment (**C**). Red lines separate genes at the *x*-axis values that correspond to *p*-value 0.01 and at the *y*-axis values that correspond to logFC of 1. The black lines represent the result of a hierarchical clustering of samples by R function heatmap.2. Auxin metabolism-related DEGs are labeled on the treatment volcano plots. Heatmap of (rlog-transformed) read counts for DEGs involved in auxin metabolism (**D**). Strict mapping of *A. thaliana* auxin metabolism genes to *B. rapa* genes was used (BBH, max length difference 0.8, identity > 40%). DEGs involved in auxin metabolism are: *YDK1* (BraA01g001510.3C), *IAA2* (BraA03g040660.3C), *IAA29* (BraA03g057220.3C), *GH3.3* (BraA09g054360.3C), *SAUR-like auxin-responsive protein family* (BraA01g003500.3C), *SAUR-like auxin-responsive protein family* (BraA01g003520.3C), *SAUR-like auxin-responsive protein family* (BraA06g015440.3C), *SAUR-like auxin-responsive protein family* (BraA07g029140.3C), *YUC3* (BraA10g003150.3C), *EIR1* (BraA03g012590.3C), *C-S-lyase* (BraA03g054450.3C), *UGT74E2* (BraA08g034260.3C).

The DEGs involved in auxin metabolism are shown in Figure 5. The genes were annotated based on their similarity to the *A. thaliana* genes (see Methods 4.5). As can be seen, treatment with IBA-Ala (Figure 5C) affected more auxin-related genes than treatments with IAA-Ala (Figure 5A) and IPA-Ala (Figure 5B). DEGs involved in auxin metabolism found after IAA-Ala treatment include the repressors *IAA2* and *IAA29* and the IAA amino acid conjugate synthetases *YDK1* (*GH3.2*) and *GH3.3*. Other DEGs involved in auxin metabolism found after IPA-Ala treatment were the *SAUR*-like auxin-responsive protein family and *YUC3*, which encode proteins involved in IAA biosynthesis. Additional DEGs involved in auxin metabolism found only after IBA-Ala treatment were *EIR1*, *C-S lyase* and *UGT74E2*.

A heatmap of the (rlog-transformed) read counts for DEGs involved in auxin metabolism is shown in Figure 5D. As can be seen, the expression of all detected genes, with the exception of *C-S lyase*, was higher under the applied treatments compared to the control. The *C-S lyase* gene, which is involved in IAA biosynthesis via the indole-3-acetaldoxime (IAOx) pathway [32], was most strongly downregulated by IPA-Ala, followed by IAA-Ala and IBA-Ala. The expression of *YDK1* was upregulated by IAA-Ala and IBA-Ala compared to IPA-Ala and the untreated control. *SAUR*-like gene families were overexpressed in all treatments compared to the control, although the intensity was different in each treatment. The *UGT74E2* and *GH3.3* genes were overexpressed in all treatments, with the highest levels following the IBA-Ala and IAA-Ala treatments, respectively. IAA-Ala caused higher expression of *YDK1* and *IAA29* compared to the other treatments.

**Figure 6:**
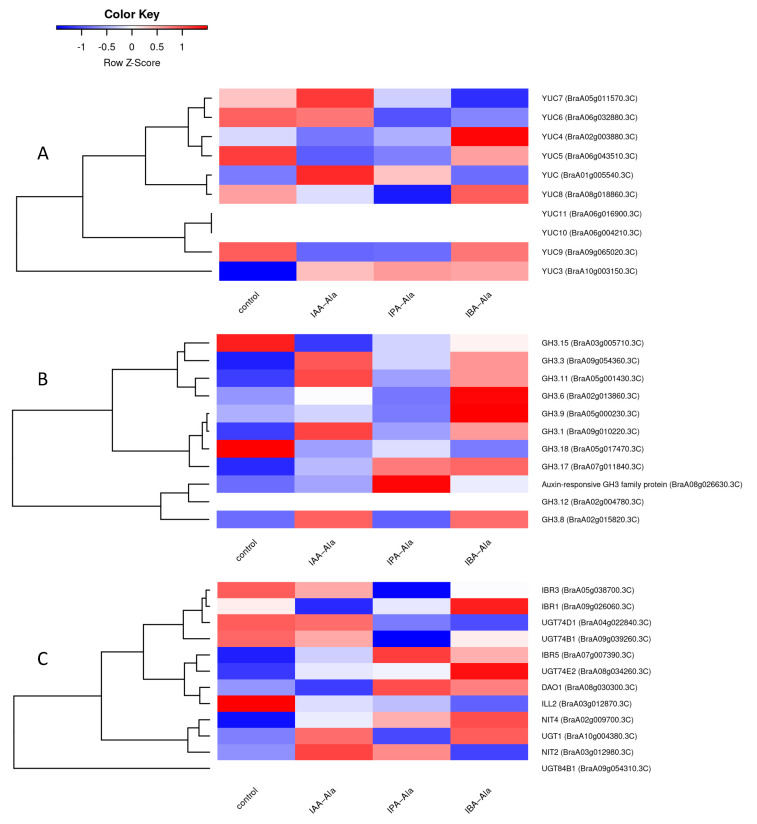
Heatmap of (rlog-transformed) read counts for *YUC* genes (**A**), *GH3* genes (**B**) and other auxin-related genes of interest (*NIT*, *IBR*, *UGT*, *DAO*) (**C**) involved in auxin metabolism scaled by row. Strict mapping of *A. thaliana* auxin metabolism genes to *B. rapa* genes was used (BBH, max length difference 0.8, identity > 40%).

All of the genes known to be involved in auxin metabolism in *A. thaliana* that were found in *B. rapa* in this work were analyzed using a heatmap (Figure S7). The effects of IBA-Ala and IPA-Ala on auxin metabolism in *B. rapa* were more similar to each other than the effects of IAA-Ala. The expression pattern of a more specific group of genes (not necessarily DEGs) involved in auxin metabolism is shown in Figure 6. Since the change in expression of most of these genes was not strong enough to be statistically significant, Figure 6 can only show general trends and/or hypotheses. For example, there appears to be a tendency for IPA-Ala to have a stronger inhibitory effect on most *YUC* genes compared to the control and other treatments. All treatments appear to have a tendency to downregulate *GH3.15* and *GH3.18* and upregulate *GH3.3*, *GH3.11*, *GH3.1* and *GH3.17* (Figure 6B). In all treatments, the genes encoding glycosyltransferases *UGT74D1* and *UGT74B1* appear to have been downregulated and *UGT74E2* upregulated (Figure 6C). *NIT2*, which is involved in the alternative IAA biosynthetic pathway (IAOx pathway), also showed a tendency to be induced in the treatments used here, except after IBA-Ala. *DAO1*, which is responsible for the oxidation of IAA and IAA-amino acid conjugates, showed a tendency towards induction in IBA-Ala and IPA-Ala treatments. The similarity of effects between the three auxin conjugate treatments may lend additional credence to the observed trends in Figure 6, but further experiments are required to confirm them.

## 3. Discussion

### 3.1. Root Growth Inhibition by Auxin Amino Acid Conjugates

The inhibitory effect of auxin amino acid conjugates IAA-Ala, IPA-Ala and IBA-Ala on Chinese cabbage root growth has been documented earlier, in a concentration range of 0.1 μM to 0.1 mM [8]. Here, we applied the same amino acid conjugates at a concentration of 10 μM to examine their effect on the auxin metabolome and transcriptome with a special focus on auxin responsive genes. Root growth inhibition caused by a 10 μM concentration of applied amino acid conjugates was in accordance with previous results [8]; IBA-Ala and IPA-Ala were shown to be more potent inhibitors of root growth compared to IAA-Ala (Figure S1). Similarly, root growth inhibitory effects for IAA conjugates were reported for *A. thaliana* [3,33].

### 3.2. Auxin Metabolome

To determine whether these conjugates have an effect on changes in auxin metabolism, the profile and content of auxin metabolites in *B. rapa* seedlings after treatment with these conjugates were investigated by UHPLC-MS/MS analysis.

The content of auxin biosynthetic precursors in *B. rapa* seedlings was relatively constant after 24 h of treatment with all conjugates, thus excluding auxin biosynthesis as a mechanism to control free IAA content under our experimental conditions. Until recently, free IAA content was thought to account for about 25% of total IAA, depending on the plant species [4]. However, considering the results of the analysis in this study, as well as previous analyses of auxin metabolism [10,34], the content of conjugates relative to free IAA is much lower than previously thought. In addition, the tryptophan content is very high (in the milligram scale) compared to most other precursors and metabolites, which has already been observed in *A. thaliana* [10].

When compared to the corresponding control, the levels of IAA-Asp and IAA-Glu, which have been identified as precursors of the catabolic pathway [4], do not change significantly when treated with the auxin conjugates. This suggests that, in our experimental setup, the mechanism of reduction of IAA by conjugating IAA to Asp and Glu was unaffected. There was a significant decrease in IAA-Asp after the IPA-Ala treatment compared to the IAA-Ala treatment. It is possible that an excess of hydrolyzed IPA cannot be converted to IAA and consequently increases the level of IAA-Asp as a potential catabolic precursor. Another scenario would involve its conjugation back to IPA-amino acid conjugates such as IPA-Asp, but we cannot confirm this at this stage due to the limitations of the analytical methods. This is in contrast to the results observed with exogenous IAA treatments [35,36], where “feeding” *A. thaliana* with high concentrations of IAA (above 5 µmol L^−1^) increased IAA conjugation to Asp and Glu and further oxidation of IAA-Asp to oxIAA-Asp and OH-IAA-Asp, while treatments with lower concentrations of exogenous IAA (below 0.5 µmol L^−1^) mainly increased oxIAA. Recent research has shed new light on auxin inactivation as a part of auxin homeostasis and has shown that the process of auxin inactivation is more complex, comprising the complete GH3-ILR1-DAO enzyme pathway [30,31]. It has been shown that IAA is first converted to IAA-amino acid conjugates (IAA-Asp and IAA-Glu) by GH3 enzymes, and then IAA-Asp and IAA-Glu can be converted back to IAA by ILR1/ILL amidohydrolases or irreversibly oxidized to oxIAA-Asp and oxIAA-Glu by DAO1 dioxygenase. oxIAA-Asp and oxIAA-Glu can subsequently be hydrolyzed by ILR1 to release inactive oxIAA. Furthermore, based on comparative feeding experiments on *A. thaliana*, *Picea abies* and *Physcomitrium patens*, Brunoni et al. [31] reported that the pathway of auxin inactivation is species-specific. At the moment, we were not able to measure all intermediates of the auxin inactivation pathway. Therefore, the metabolites oxIAA and oxIAA-glc were identified as the major catabolic products under our experimental conditions. It has been previously shown that oxIAA has a low biological activity, and it has also been found to vary at the cellular level in the root tip [37]. Tissue-specific fluctuations (as well as variations between cell types) leading to the formation of auxin gradients should also be considered in *B. rapa* seedlings, but in our work we focused on auxin content at the level of the whole seedling. All treatments showed a tendency to increase oxIAA content compared to the control, although IBA-Ala did not show statistical significance as observed for IPA-Ala and IAA-Ala. Further, oxIAA-glc metabolite was increased in all treatments, with the IBA-Ala treatment being statistically significant. Based on current knowledge, excess IAA produced by IAA-Ala hydrolysis is metabolized into catabolic metabolites oxIAA and/or oxIAA-glc to maintain acceptable auxin levels for plant survival [30,35]. IBA released by IBA-Ala hydrolysis can also be converted to IAA by β-oxidation as previously described [11,12], which resulted in increased levels of oxIAA and oxIAA-glc in seedlings treated with IBA-Ala. So far, we have no experimental evidence for the conversion of IPA to IAA, so an increased content of oxIAA after IPA-Ala treatment requires additional experiments to explain this observation. Interestingly, conjugates IAA-Ala and IAA-Leu were only identified in *B. rapa* seedlings when treated with IAA-Ala. Nevertheless, the content of IAA-Ala and IAA-Leu is on the nanogram scale, as was the case with the conjugates IAA-Asp and IAA-Glu. This could indicate that an excess of IAA could also be conjugated to other amino acid conjugates such as IAA-Leu in addition to IAA-Asp and IAA-Glu.

Free forms of the auxins IPA and IBA were only detected in IPA-Ala and IBA-Ala feeding experiments (Figure 1), which is direct evidence for the hydrolyzes of IPA-Ala and IBA-Ala conjugates in vivo. According to our results, the content of IPA is about 6 µg g^−1^ dw, while the content of IBA is about 1.2 µg g^−1^ dw, and IAA is 0.12 µg g^−1^ dw, directly reflecting the reported increased catalytic efficiency and substrate specificity of auxin amino acid conjugate-hydrolases towards IPA-Ala and IBA-Ala compared to IAA-Ala [8]. IPA and IBA were previously described as very potent auxins that inhibited root growth. Thus, free IBA and IPA in the IBA-Ala and IPA-Ala treatments, respectively, in addition to free IAA, are consistent with the stronger inhibition of root growth measured after these treatments compared to the IAA-Ala treatment and the corresponding control.

### 3.3. Transcriptome Analysis

Specific alterations in gene transcription occurred in *B. rapa* seedlings after treatments with all auxin amino acid conjugates. In comparison to the untreated control, the treatments exhibited comparable patterns and resulted in the overexpression of several genes (Figure S3). Treatments with long-chained conjugates (IBA-Ala and IPA-Ala) showed more similar effects on the transcriptome compared to IAA-Ala and control conditions (Figure 2), consistent with their similarities in inhibition of root growth and disruption of auxin metabolism. IBA-Ala caused many more DEGs (807) compared to IPA-Ala (417) and IAA-Ala (371) (Figure 3 and Figure 4). Due to the lack of literature describing transcriptome analyses after treatments with auxin conjugates, we cannot compare our data with others. However, there are some data on the effects of auxin treatment on the transcriptomes of *Eucalyptus* [38], *A. thaliana* [39] and *B. napus* [40]. IBA feeding experiments on *Eucalyptus* resulted in changes in the expression of 702 transcripts [38]. This represents a high number of DEGs and is consistent with our data from the IBA-Ala treatment.

**Figure 7:**
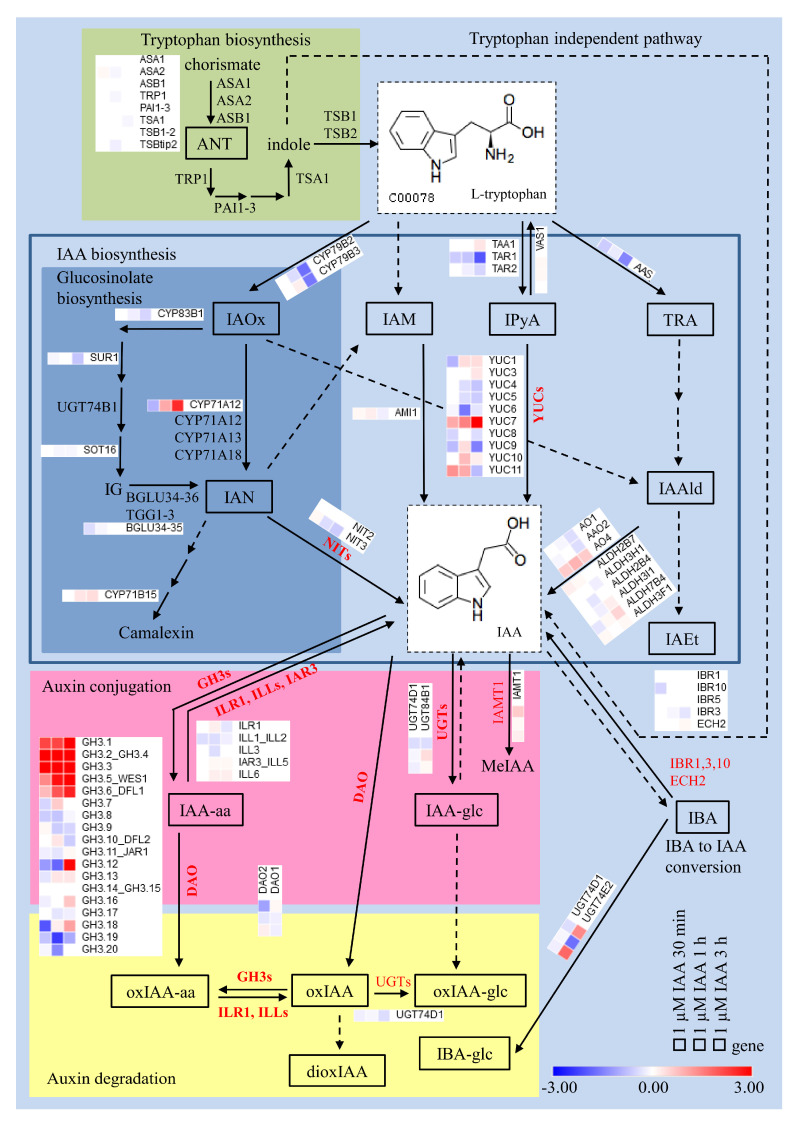
Scheme of auxin homeostasis in *A. thaliana* by expression of genes involved in early (30 min), medium (1 h) and late response (3 h) to IAA treatment (*c* = 1 µmol L^−1^). Microarray data set from AtGenExpress Hormone Series [43] for genes involved in auxin metabolism in *A. thaliana* was used from eFP expression browser [41]. Model is edited based on the recent data of Hayaski et al. [30] and Brunoni et al. [31]. The expression is presented as log_2_ fold change. The heat maps were generated with the Morpheus software (https://software.broadinstitute.org/morpheus/, accessed on 1 June 2022).

We were particularly interested in how treatments with amino acid conjugates affected auxin-related genes. Volcano plots (Figure 5) show that there are auxin-related genes with significant changes in expression (i.e., DEGs), especially under IBA-Ala, compared to IPA-Ala and finally IAA-Ala treatments. All of the DEGs detected, with the exception of C-S-lyase, were upregulated depending on the treatments, but to varying degrees. A more detailed analysis was performed to compare the effects of the treatments on various *YUC* genes (Figure 6A), *GH3* genes (Figure 6B) and various genes of interest such as *UGT*, *IBR*, etc. (Figure 6C). Since there is no information in the literature on the effects of auxin amino acid conjugates on the auxin transcriptome, we analyzed transcriptomic data in *A. thaliana* after 1 µM IAA treatment available in the database (*A. thaliana* eFP Browser (http://bar.utoronto.ca/, accessed on 1 June 2022) [41,42]. The positions of genes in the auxin metabolic pathway and their expression patterns after IAA treatments are shown in Figure 7 to compare these with our data.

The proteins encoded by the *YUC* genes are responsible for the conversion of the precursor IPyA into IAA. All conjugate treatments show a tendency to inhibit the expression of the *YUC5*, *YUC6* and *YUC9* genes, while some treatments cause inhibition of *YUC4* (IAA-Ala, IPA-Ala), *YUC7* (IPA-Ala, IBA-Ala) and *YUC8* (IAA-Ala, IPA-Ala) (Figure 6A), similar to the free auxin treatment (Figure 7). Only *YUC3* showed higher expression in all treatments compared to the control, which also corresponds to the longest treatment with free IAA (3 h) (Figure 7). Notably, the *YUC7* gene is overexpressed only in the treatment with IAA-Ala compared to the others, which is consistent with the publicly available data from the treatments with free IAA (Figure 7). When compared to the control and IAA-Ala treatments, the IBA- and IPA-Ala treatments tended to have a higher inhibitory effect on the majority of *YUC* genes. In agreement with this result, IBA-Ala and IPA-Ala caused a significant decrease in free IAA and a more pronounced inhibition of root growth compared to the control and IAA-Ala treatments. Remarkably, all treatments with auxin conjugates up-regulated the expression of *GH3.3*, *GH3.1* and *GH3.17* (Figure 6B), similar to the treatment with free IAA, which induced their expression together with *GH3.1* to *GH3.6* (Figure 7). At least for Arabidopsis, the substrates for most of the different GH3 proteins have been elucidated. The GH3.3 as well as the other GH3 proteins 3.1 to 3.6 are involved in the conjugation of IAA and oxIAA to amino acids [31,44]. The GH3.12 protein is involved in the biosynthesis of salicylic acid [45] and its expression was not detected in our system (Figure 6B). Similar observations of the inducible effects of IAA treatment on *GH3* genes have been reported by other authors [39,46]. *GH3.2 (YDK1)* was significantly induced by the treatments applied here (Figure 5), and some *GH3* genes showed a tendency to induce their expression after treatments with auxin conjugates (Figure 6B,) although this had no effect on the level of identified auxin amino acid conjugates (IAA-Asp and IAA-Glu) in *B. rapa* seedlings after the treatments. Based on the data of Brunoni et al. [31], the group II GH3 enzymes, AtGH3.1, AtGH3.2, AtGH3.3, AtGH3.5, AtGH3.6 and AtGH3.17, showed activity with oxIAA; AtGH3.2, AtGH3.3 and AtGH3.5 conjugated oxIAA preferentially with Asp, whereas AtGH3.1 and AtGH3.6 conjugated oxIAA exclusively with Asp and AtGH3.17 with Glu. Furthermore, AtGH3.2 conjugates oxIAA with Leu and Phe. Because we were unable to measure oxIAA-amino acid conjugates at the time of these experiments, additional studies are needed to confirm the presence of potential oxidized auxin conjugates under IAA-Ala, IPA-Ala and IBA-Ala treatments.

*UGT74E2* showed a tendency for expression induction after the treatments but did not significantly change the level of IAA-glc (Figure 1 and Figure 6C). *IBR* genes, which are thought to be responsible for the conversion of IBA to IAA, were mostly inhibited or remained unchanged by the treatments. However, *IBR1* shows a tendency to increase expression upon IBA-Ala treatment (Figure 6C). The detection of IPA and IBA in feeding experiments with IPA-Ala and IBA-Ala is direct evidence for the hydrolysis of these conjugates in vivo. Consistent with the qPCR results of Savić et al. [8], there is a trend for the *ILL2* gene, which encodes a conjugate hydrolase, to be suppressed after treatments as compared to the control (Figure S7). Consequently, treatment with free IAA downregulated auxin amido-hydrolases (*ILR*, *ILL*, and *IAR3*) (Figure 7).

Our transcriptomic data are largely consistent with transcriptomic data obtained from free IAA treatments in Arabidopsis and confirm that free auxins released by hydrolysis of the conjugates used here (IPA, IBA and IAA) are responsible for gene regulation, auxin homeostasis and consequently root growth inhibition. Further experiments with other auxin conjugates are necessary to elucidate their role in plant development.

## 4. Materials and Methods

### 4.1. Plant Materials

Seeds of Chinese cabbage (*Brassica rapa* L. ssp. *pekinensis* (Lour.) Hanelt cv. Cantonner Witkrop) were purchased from ISP International Seed Processing GmbH, Quedlinburg, Germany. Following sterilization, seeds were germinated and the 1-day-old seedlings were placed on 1% agar plates, supplemented with the hormones idole-3-acetyl-L-alanine (IAA-Ala), indole-3-propionyl-L-alanine (IPA-Ala) and indole-3-butyryl-L-alanine (IBA-Ala) in 0.01 mM concentration as described earlier [8]. IAA-Ala, IPA-Ala and IBA-Ala were synthesized as previously published. Seedlings on hormone-free agar plates were used as a control. Following incubation of the seedlings under continuous light for a further 24 h at 22 °C, the increase in root lengths was monitored. Plates were then scanned (Canon scanner) and root images analyzed with the program ImageJ v.1.53s (Figure S1B). Two-day-old seedlings used for analyses of auxin were lyophilized.

### 4.2. Auxin Quantification

IAA metabolite profiling was performed as described earlier [10] using 2 mg of lyophilized material. Briefly, freeze-dried samples were homogenized using a MixerMill (Retsch GmbH, Haan, Germany) and extracted in 1 mL of ice-cold 50 mM sodium phosphate buffer (pH 7.0) containing 0.1% sodium diethyldithiocarbamate and the following deuterium- or ^13^C-labeled internal standards: [^2^H_4_]ANT, [^13^C_6_]IAA, [^2^H_5_]IAM, [^2^H_5_]IAOx, [^2^H_4_]IPyA, [^13^C_6_]oxIAA, [^2^H_4_]TRA, [^13^C_6_]IAA-Asp, [^13^C_6_]IAA-Glu, [^13^C_6_]IAA-glc, [^13^C_6_]oxIAA-glc, [^2^H_5_,^15^N]IAA-Ala, [^2^H_5_,^15^N]IAA-Leu, [^13^C_6_,^15^N]IBA, [^2^H_2_]IPA (5 pmol per sample); [^2^H_4_]IAN (10 pmol per sample) and [^2^H_5_]TRP (50 pmol per sample). Indole-3-acetic acid and other indole compounds were obtained from OlChemIm (Olomouc, Czech Republic). The resulting extract was divided into two equal volumes. The first volume was acidified with 1 M HCl to pH 2.7 and purified by solid phase extraction (SPE) using OasisTM HLB columns (30 mg, 1 mL; Waters, Milford, MA, USA). The second volume of the supernatant was derivatized by incubation with 3 mL of a 0.25 M cysteamine solution (pH 8.0) for 1 h at room temperature. The derivatized sample was then purified as described above. After evaporation under reduced pressure, samples were analyzed using Acquity UHPLC^TM^ (Waters, USA) linked to a triple quadrupole mass detector (Xevo TQ MSTM; Waters, USA). Instrument settings were as previously published for auxin profiling [10]. Four independent replicate analyses were performed for each hormone.

### 4.3. Statistical Analysis

The results of each analysis were compared using analysis of variance (ANOVA) with Tukey’s HSD Post Hoc test, as implemented in the XLSTAT software package v.2022.3; differences between exposure treatments and the corresponding controls were considered statistically significant if *p* < 0.05. Each reported data point represents the average of four replicates (*n* = 4) unless stated otherwise. One replicate for hormone analysis was defined as one agar plate which contained approximately 10 seedlings.

### 4.4. RNA-Seq Analysis

Total RNA was isolated from 100 mg of whole seedlings, which were homogenized in a Mixer Mill MM400 (Retsch GmbH, Germany) in three cycles of 1 min at 30 Hz with zirconium oxide beads (Next Advance, USA). To each pulverized sample, 1 mL of RNAzol (Sigma Aldrich, St. Louis, MI, USA) was added, and after centrifugation, the same volume of ethanol (100%) was added to the supernatants, which were further processed through the Direct-zolTM RNA MiniPrep kit (Zymo Research, Irvine, CA, USA) according to the manufacturer’s instructions. The eluted total RNA samples were checked for concentration on Nanodrop 2000. Total RNA (two biological replicates per treatment) was processed and sequenced by Vienna BioCenter Core Facilities (VBCF NGS, Vienna, Austria). Single reads of length ~50 bp were obtained from the sequencing facility, processed and analyzed for differential expression between the samples. Raw reads showed an unstable base composition in the first 9 bp, as visualized by FastQC. STAR v.2.7.1a [47] was used to clip the first 9 bp of the reads (improving the mismatch rate per base statistic), as well as to align them with the reference genome. For the reference genome and GO annotations, we used the *Brassica rapa* Chiifu V3.0 obtained from the BRAD database [48]. Counts of reads aligning with genes were obtained using htseq-count v.1.99.2 [49]. Raw counts were used for differential expression analysis using deseq2 v.1.34.0 [50]. IAA-Ala, IBA-Ala and IPA-Ala treatments were processed and compared to the untreated control independently. The deseq2 was run in Rstudio v.2021.09.02 Build 382, running R v.4.2.0. Genes above the (FDR-adjusted) *p*-value of 0.01 were pronounced differentially expressed genes (DEGs). GSEA analyses were performed using the GSEA function of the clusterProfiler package v.4.4.4 on the genes ordered by their shrunken log2FC values [51]. The ancestor GO nodes were obtained using the buildGOmap function of the same package. RNA-seq data were deposited to NCBI and are available at https://www.ncbi.nlm.nih.gov/geo/query/acc.cgi?acc=GSE234868.

### 4.5. Mapping of B. rapa Genes to A. thaliana Genes and Gene Annotations

The genes of *B. rapa* were mapped to genes of *A. thaliana* for easier evaluation and analysis of results using blastp v.2.9.0+ from the NCBI BLAST+ package [52]. The strict mapping (BBH in the text) was designed to conservatively detect probable orthologs; the mapping criterion was that the proteins from the two species are the best bidirectional blastp hits, with differences in length < 80% and % identity ≥ 40%. The less-strict mapping (UBH in the text) followed the criterion that the *A.thaliana* candidate is the best unidirectional blast hit of the querying *B. rapa* genes, with a bitScore of at least 50 and % identity ≥ 20%. *A. thaliana* gene descriptions were downloaded from the TAIR database [53].

## 5. Conclusions

Some recently published data suggest that auxin conjugates, in addition to their storage or detoxification function, also play a novel role in plant development and stress responses [20,21]. Furthermore, it has been shown that auxin conjugates can be used as potent rooting agents, especially in hard rooting species [7]. Therefore, it is necessary to shed light on the specific new roles of auxin conjugates and the mechanisms of their action. This research focuses on elucidating some details about the mode of action of the three auxin conjugates IAA-Ala, IBA-Ala and IPA-Ala, which were applied to *B. rapa* seedlings. We identified free forms of auxin, IPA and IBA in IPA-Ala and IBA-Ala feeding experiments, which is direct evidence for the hydrolysis of IPA-Ala and IBA-Ala conjugates in vivo. Free IBA and IPA released after the treatments with their respective conjugates are consistent with the stronger inhibition of root growth compared to the IAA-Ala treatment and the control. Transcriptomic data confirmed that IBA-Ala and IPA-Ala caused more disruption in gene expression compared to IAA-Ala treatment (807, 417 and 371 DEGs, respectively). The DEGs directly related to auxin metabolism are comparable to the data gathered for IAA feeding experiments with *A. thaliana*. We can conclude that the majority of treatments, particularly with IBA- and IPA-Ala, tend to inhibit the expression of *YUC* genes encoding proteins responsible for auxin synthesis and induce the expression of *GH3* and *UGT74E2* genes, whose products are responsible for the reversible or irreversible conjugation. These observations are in accordance with our metabolomics data.

## Data Availability

RNA-seq data are available at https://www.ncbi.nlm.nih.gov/geo/query/acc.cgi?acc=GSE234868.

## Supplementary Materials

The following supporting information can be downloaded at: https://www.mdpi.com/article/10.3390/ijms25010447/s1.
