# Supplementary material for: Application of Long-Chained Auxin Conjugates Influenced Auxin Metabolism and Transcriptome Response in Brassica rapa L. ssp. pekinensis
Source: Int J Mol Sci. 2023 Dec 28;25(1):447. doi: 10.3390/ijms25010447 (PMC10778880; doi:10.3390/ijms25010447)
Supplement: Supplementary file 1 [file ijms-25-00447-s001.zip › Suplementary figures_rev_12_2023.pdf]

Application of long-chained auxin conjugates influenced auxin metabolism and transcriptome response in *Brassica rapa* L. ssp. *pekinensis*

Ana Smolko[1,†], Jelena Repar[1,†], Marija Matković[2], Iva Pavlović[3], Aleš Pěnčík[3], Ondřej Novák[3], Jutta Ludwig-Müller[4] and Branka Salopek-Sondi[1,*]

Supplementary Figures

A

B

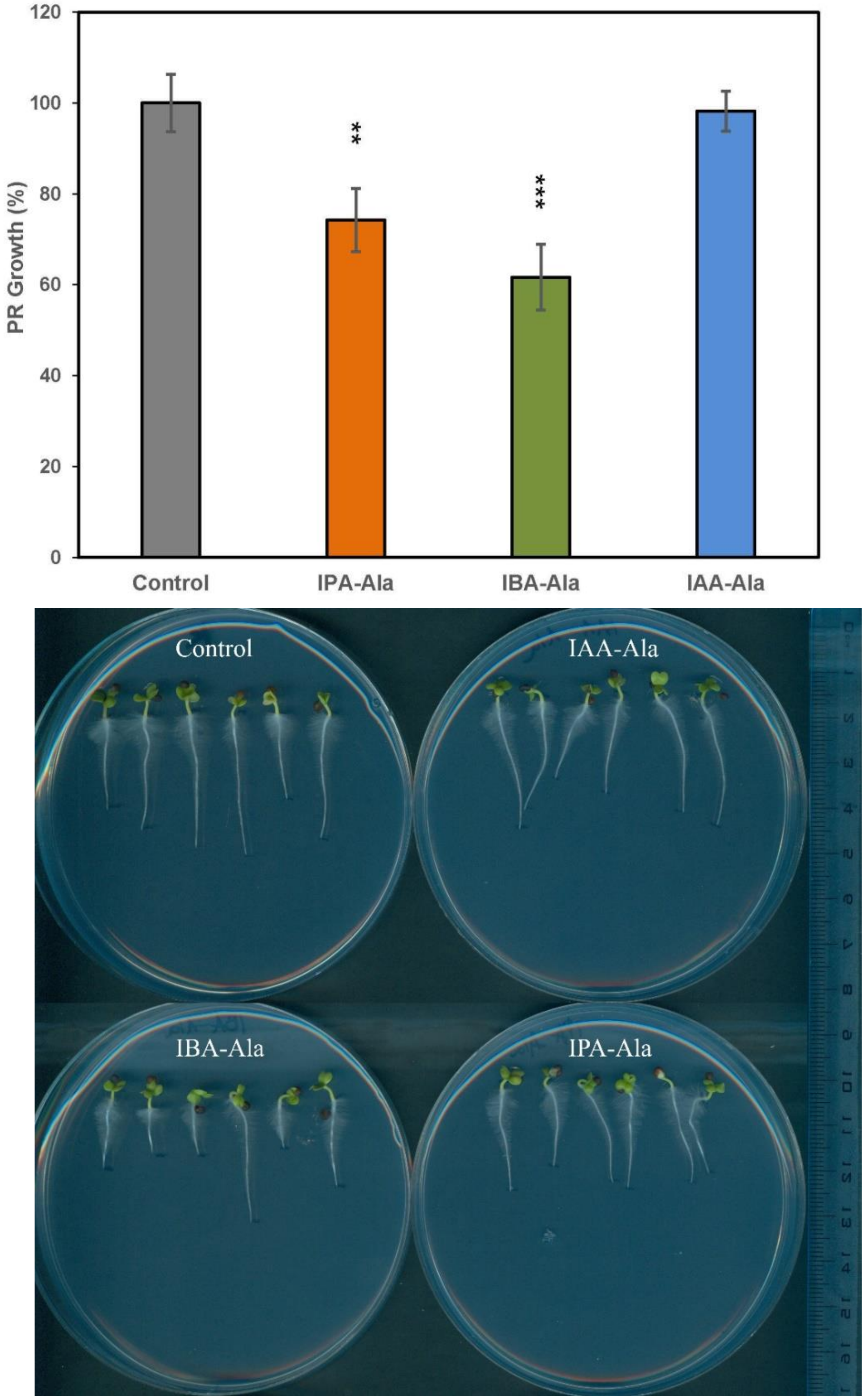

Figure S1. Average primary root (PR) length per seedling for the *Brassica rapa* seedlings under control conditions (1% agar only) versus the same media supplemented with 10 µM IAA-Ala, 10 µM IPA-Ala or 10 µM IBA-Ala (A) and image of two-days old seedlings upon treatments used for metabolomics and transcriptomic analysis (B). Data are presented as mean ± SE (n=18). Asterisk (*) indicates significant differences with respect to the control growth conditions (Student's t test, * presents P < 0.05, ** P< 0.01, *** P<0.001).

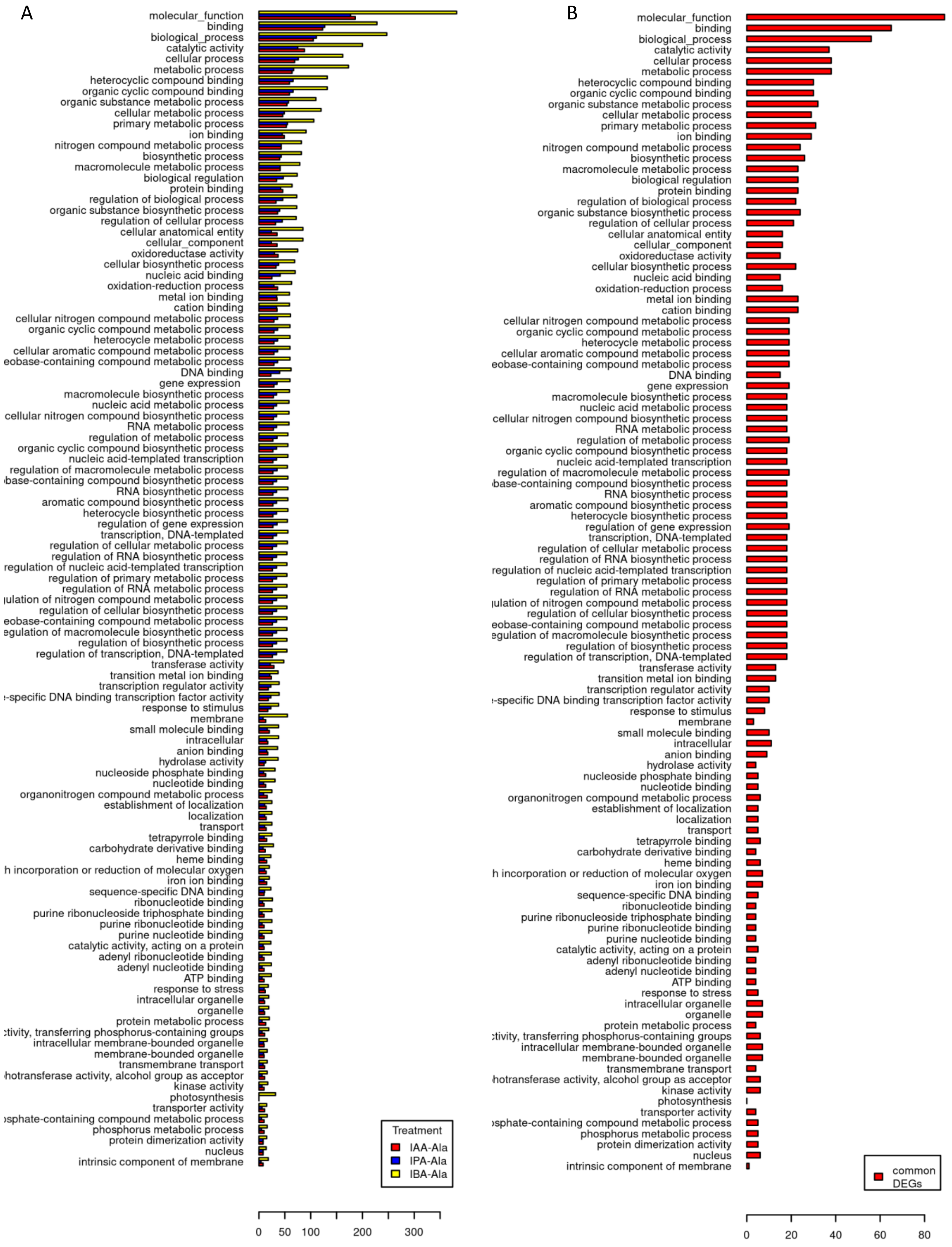

Figure S2. Gene counts per treatment per GO category (A) and counts of DEGs common to all treatments per GO category (B). One gene may belong to several GO categories. Only GO categories with 30 or more genes are included.

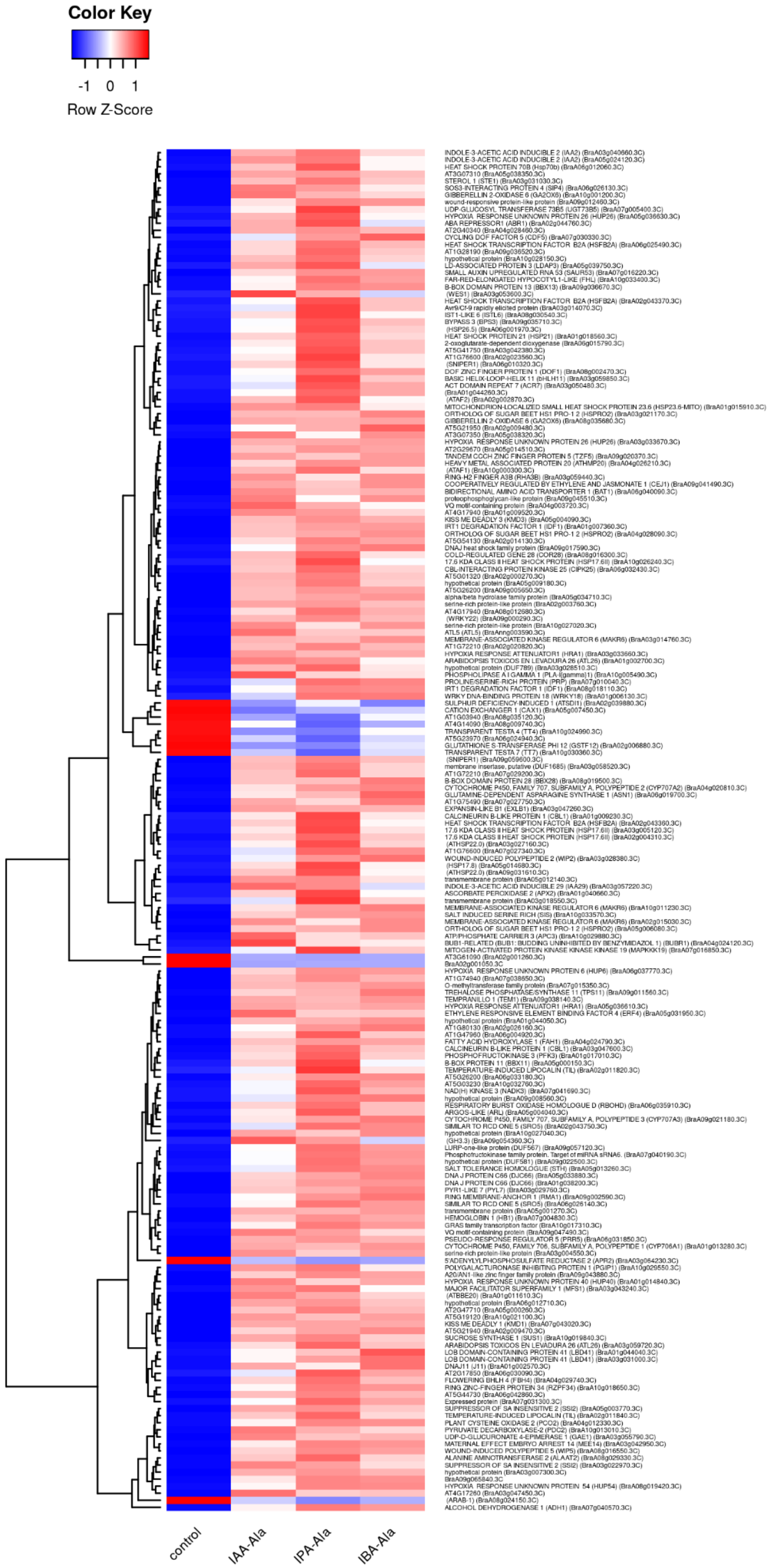

Figure S3. Heatmap of mean (rlog transformed) read counts for DEGs implicated in all 3 treatments, scaled by row. The *B. rapa* genes have been mapped to the *A. thaliana* homologs. Genes are represented by their common names in *A. thaliana*, or descriptions where common names were unavailable, as well as the *B. rapa* identifier (end of string). Less strict mapping of *A. thaliana* genes to *B. rapa* genes was used (UBH, min bitscore 50, min identity>20%).

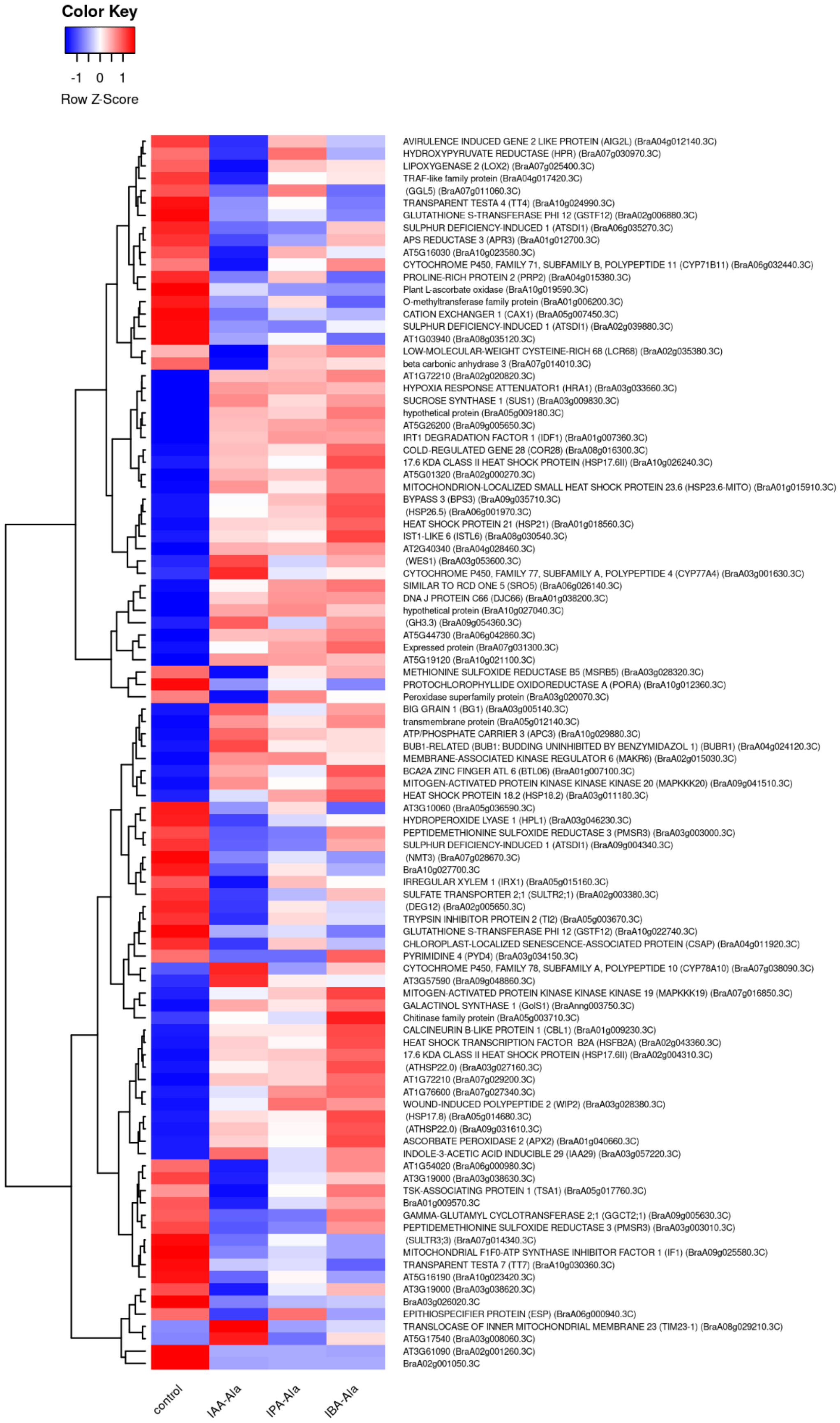

Figure S4. Heatmap of (rlog transformed) read counts for 50 genes with lowest logFCshrinked and 50 genes with highest logFCshrinked after the IAA-Ala treatment, scaled by row. The *B. rapa* genes have been mapped to the *A. thaliana* homologs. Genes are represented by their common names in *A. thaliana*, or descriptions where common names were unavailable, as well as the B. rapa identifier (end of string). Less strict mapping of *A. thaliana* genes to *B. rapa* genes was used (UBH, min bitscore 50, min identity>20%).

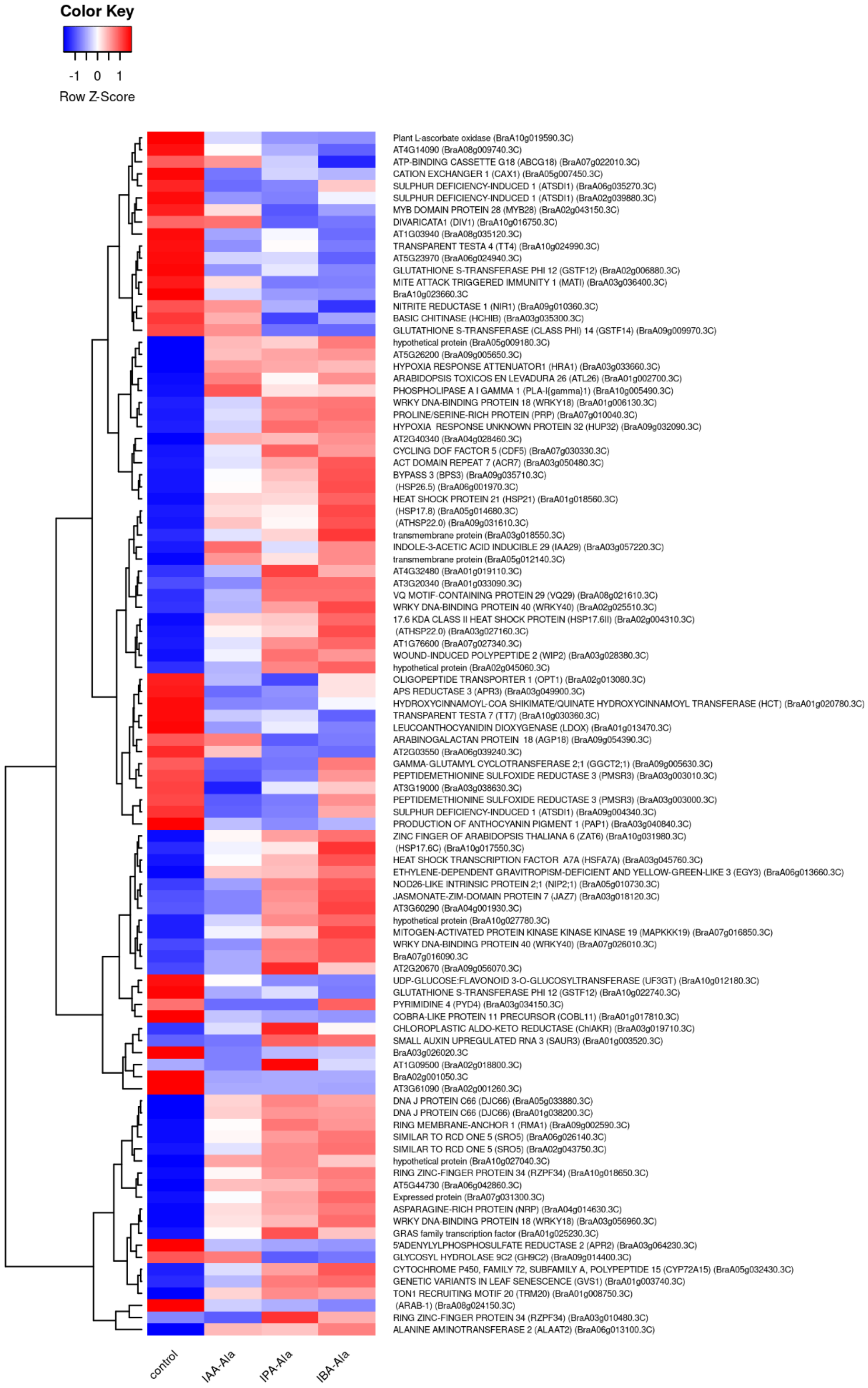

Figure S5. Heatmap of (rlog transformed) read counts for 50 genes with lowest logFCshrinked and 50 genes with highest logFCshrinked after the IPA-Ala treatment, scaled by row. The B. rapa genes have been mapped to the A. thaliana homologs. Genes are represented by their common names in A. thaliana, or descriptions where common names were unavailable, as well as the B. rapa identifier (end of string). Less strict mapping of A. thaliana genes to B. rapa genes was used (UBH, min bitscore 50, min identity>20%).

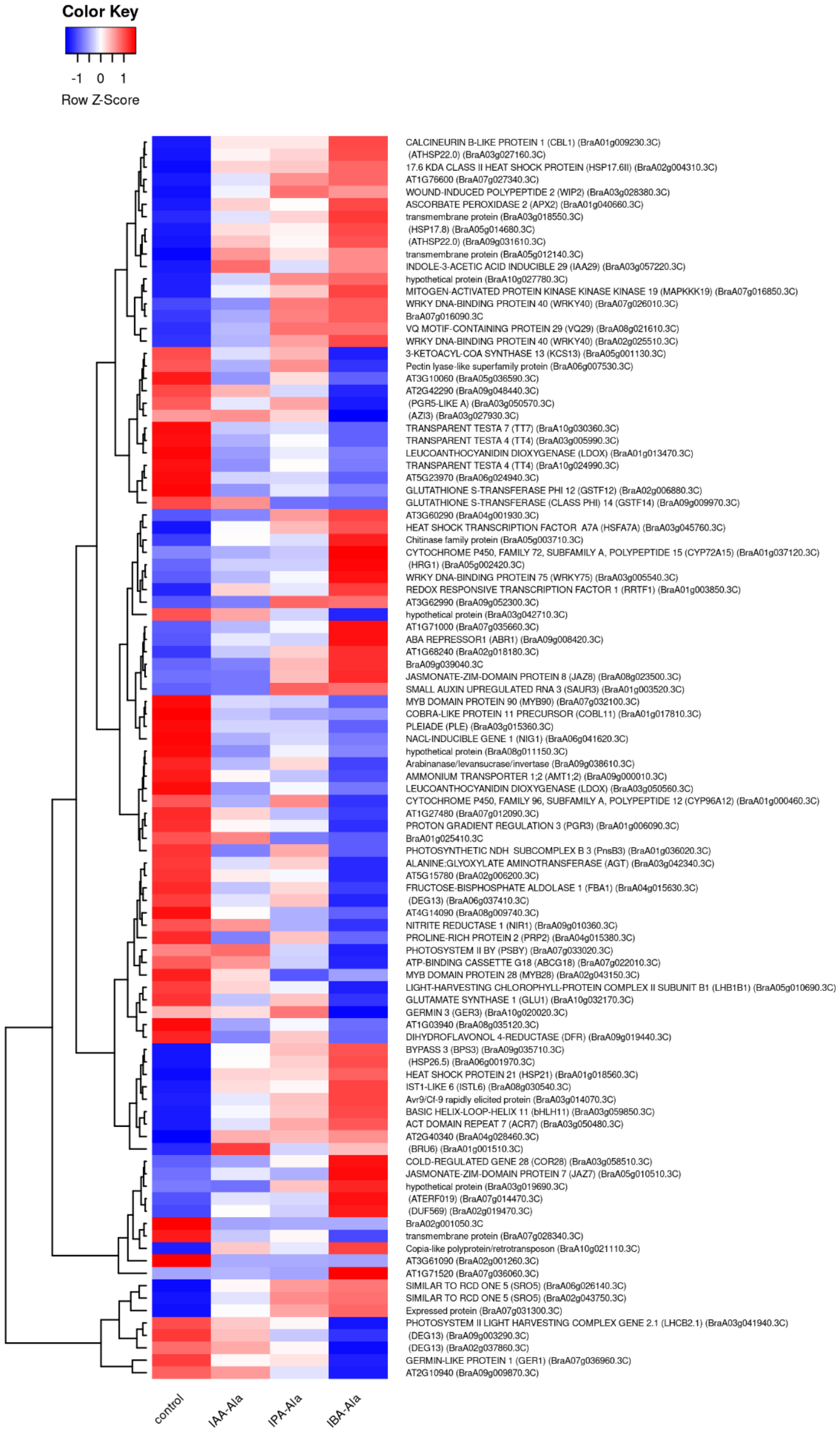

Figure S6. Heatmap of (rlog transformed) read counts for 50 genes with lowest logFCshrinked and 50 genes with highest logFCshrinked after the IBA-Ala treatment, scaled by row. The *B. rapa* genes have been mapped to the *A. thaliana* homologs. Genes are represented by their common names in *A. thaliana*, or descriptions where common names were unavailable, as well as the *B. rapa* identifier (end of string). Less strict mapping of *A. thaliana* genes to *B. rapa* genes was used (UBH, min bitscore 50, min identity>20%).

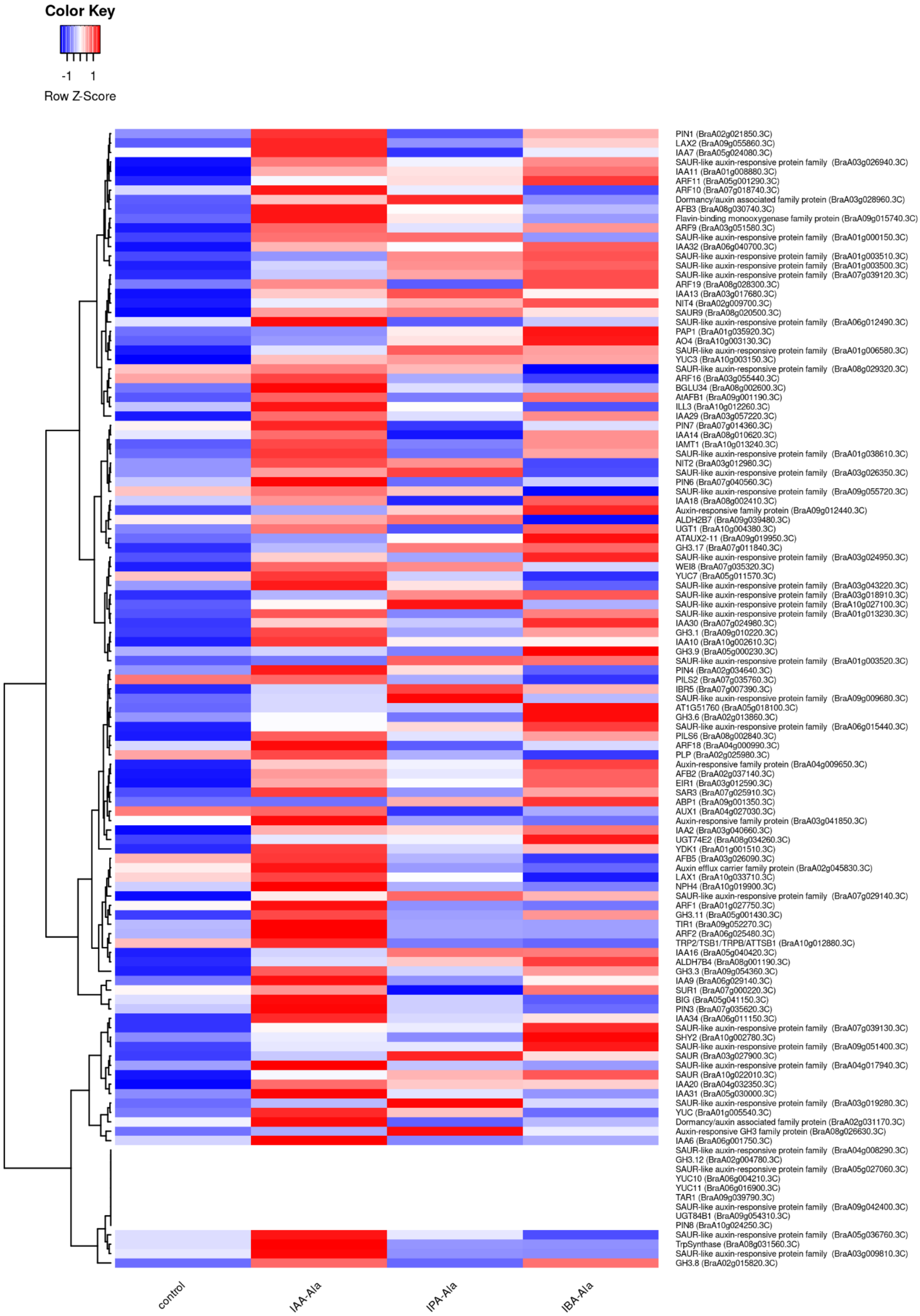

Color Key
-1
1
Row Z-Score
PIN1 (BraA02g021850.3C)
LAX2 (BraA09g055860.3C)
IAA7 (BraA05g024080.3C)
SAUR-like auxin-responsive protein family (BraA03g026940.3C)
IAA11 (BraA01g008880.3C)
ARF11 (BraA05g001290.3C)
ARF10 (BraA07g018740.3C)
Dormancy/auxin associated family protein (BraA03g028960.3C)
AFB3 (BraA08g030740.3C)
Flavin-binding monooxygenase family protein (BraA09g015740.3C)
ARF9 (BraA03g051580.3C)
SAUR-like auxin-responsive protein family (BraA01g000150.3C)
IAA32 (BraA06g040700.3C)
SAUR-like auxin-responsive protein family (BraA01g003510.3C)
SAUR-like auxin-responsive protein family (BraA01g003500.3C)
SAUR-like auxin-responsive protein family (BraA07g039120.3C)
ARF19 (BraA08g028300.3C)
IAA13 (BraA03g017680.3C)
NIT4 (BraA02g009700.3C)
SAUR9 (BraA08g020500.3C)
SAUR-like auxin-responsive protein family (BraA06g012490.3C)
PAP1 (BraA01g035920.3C)
AO4 (BraA10g003130.3C)
SAUR-like auxin-responsive protein family (BraA01g006580.3C)
YUC3 (BraA10g003150.3C)
SAUR-like auxin-responsive protein family (BraA08g029320.3C)
ARF16 (BraA03g055440.3C)
BGLU34 (BraA08g002600.3C)
AtAFB1 (BraA09g001190.3C)
ILL3 (BraA10g012260.3C)
IAA29 (BraA03g057220.3C)
PIN7 (BraA07g014360.3C)
IAA14 (BraA08g010620.3C)
IAMT1 (BraA10g013240.3C)
SAUR-like auxin-responsive protein family (BraA01g038610.3C)
NIT2 (BraA03g012980.3C)
SAUR-like auxin-responsive protein family (BraA03g026350.3C)
PIN6 (BraA07g040560.3C)
SAUR-like auxin-responsive protein family (BraA09g055720.3C)
IAA18 (BraA08g002410.3C)
Auxin-responsive family protein (BraA09g012440.3C)
ALDH2B7 (BraA09g039480.3C)
UGT1 (BraA10g004380.3C)
ATAUX2-11 (BraA09g019950.3C)
GH3.17 (BraA07g011840.3C)
SAUR-like auxin-responsive protein family (BraA03g024950.3C)
WEI8 (BraA07g035320.3C)
YUC7 (BraA05g011570.3C)
SAUR-like auxin-responsive protein family (BraA03g043220.3C)
SAUR-like auxin-responsive protein family (BraA03g018910.3C)
SAUR-like auxin-responsive protein family (BraA10g027100.3C)
SAUR-like auxin-responsive protein family (BraA01g013230.3C)
IAA30 (BraA07g024980.3C)
GH3.1 (BraA09g010220.3C)
IAA10 (BraA10g002610.3C)
GH3.9 (BraA05g000230.3C)
SAUR-like auxin-responsive protein family (BraA01g003520.3C)
PIN4 (BraA02g034640.3C)
PILS2 (BraA07g035760.3C)
IBR5 (BraA07g007390.3C)
SAUR-like auxin-responsive protein family (BraA09g009680.3C)
AT1G51760 (BraA05g018100.3C)
GH3.6 (BraA02g013860.3C)
SAUR-like auxin-responsive protein family (BraA06g015440.3C)
PILS6 (BraA08g002840.3C)
ARF18 (BraA04g000990.3C)
PLP (BraA02g025980.3C)
Auxin-responsive family protein (BraA04g009650.3C)
AFB2 (BraA02g037140.3C)
EIR1 (BraA03g012590.3C)
SAR3 (BraA07g025910.3C)
ABP1 (BraA09g001350.3C)
AUX1 (BraA04g027030.3C)
Auxin-responsive family protein (BraA03g041850.3C)
IAA2 (BraA03g040660.3C)
UGT74E2 (BraA08g034260.3C)
YDK1 (BraA01g001510.3C)
AFB5 (BraA03g026090.3C)
Auxin efflux carrier family protein (BraA02g045830.3C)
LAX1 (BraA10g033710.3C)
NPH4 (BraA10g019900.3C)
SAUR-like auxin-responsive protein family (BraA07g029140.3C)
ARF1 (BraA01g027750.3C)
GH3.11 (BraA05g001430.3C)
TIR1 (BraA09g052270.3C)
ARF2 (BraA06g025480.3C)
TRP2/TSB1/TRPB/ATTSB1 (BraA10g012880.3C)
IAA16 (BraA05g040420.3C)
ALDH7B4 (BraA08g001190.3C)
GH3.3 (BraA09g054360.3C)
IAA9 (BraA06g029140.3C)
SUR1 (BraA07g000220.3C)
BIG (BraA05g041150.3C)
PIN3 (BraA07g035620.3C)
IAA34 (BraA06g011150.3C)
SAUR-like auxin-responsive protein family (BraA07g039130.3C)
SHY2 (BraA10g002780.3C)
SAUR-like auxin-responsive protein family (BraA09g051400.3C)
SAUR (BraA03g027900.3C)
SAUR-like auxin-responsive protein family (BraA04g017940.3C)
SAUR (BraA10g022010.3C)
IAA20 (BraA04g032350.3C)
IAA31 (BraA05g030000.3C)
SAUR-like auxin-responsive protein family (BraA03g019280.3C)
YUC (BraA01g005540.3C)
Dormancy/auxin associated family protein (BraA02g031170.3C)
Auxin-responsive GH3 family protein (BraA08g026630.3C)
IAA6 (BraA06g001750.3C)
SAUR-like auxin-responsive protein family (BraA04g008290.3C)
GH3.12 (BraA02g004780.3C)
SAUR-like auxin-responsive protein family (BraA05g027060.3C)
YUC10 (BraA06g004210.3C)
YUC11 (BraA06g016900.3C)
TAR1 (BraA09g039790.3C)
SAUR-like auxin-responsive protein family (BraA09g042400.3C)
UGT84B1 (BraA09g054310.3C)
PIN8 (BraA10g024250.3C)
SAUR-like auxin-responsive protein family (BraA05g036760.3C)
TrpSynthase (BraA08g031560.3C)
SAUR-like auxin-responsive protein family (BraA03g009810.3C)
GH3.8 (BraA02g015820.3C)
control
IAA-Ala
IPA-Ala
IBA-Ala

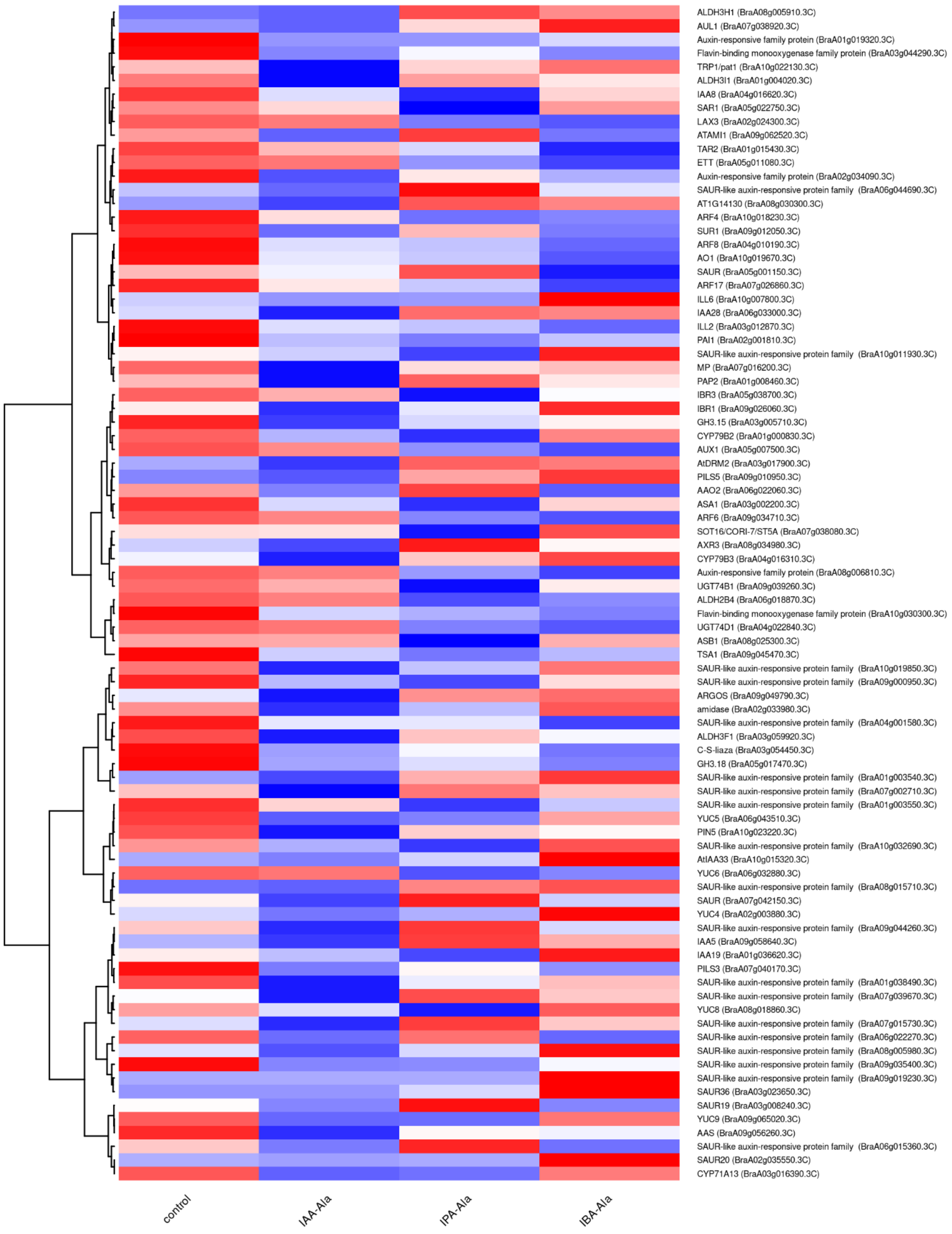

Figure S7. Heatmap of (rlog transformed) mean read counts for genes involved in auxin metabolism with count values scaled by row. The gene list has been split in two for readability. The *B. rapa* genes have been mapped to the *A. thaliana* homologs. Genes are represented by their common names in *A. thaliana*, or descriptions where common names were unavailable. Strict mapping of *A. thaliana* auxin metabolism genes to *B. rapa* genes was used (BBH, max length difference 0.8, identity>40%).
